# When Good Turns Bad: Regulation of Invasion and Metastasis by ErbB2 Receptor Tyrosine Kinase

**DOI:** 10.3390/cells3010053

**Published:** 2014-01-27

**Authors:** Ditte Marie Brix, Knut Kristoffer Bundgaard Clemmensen, Tuula Kallunki

**Affiliations:** Unit of Cell Death and Metabolism, Danish Cancer Society Research Center, Danish Cancer Society, Strandboulevarden 49, DK-2100 Copenhagen, Denmark; E-Mails: dittemb@cancer.dk (D.M.B.); knucle@cancer.dk (K.K.B.C.)

**Keywords:** cathepsin B, cathepsin L, matrix metalloprotease, p95 ErbB2, p21-activated protein kinase, transforming growth factor β

## Abstract

Overexpression and activation of ErbB2 receptor tyrosine kinase in breast cancer is strongly linked to an aggressive disease with high potential for invasion and metastasis. In addition to inducing very aggressive, metastatic cancer, ErbB2 activation mediates processes such as increased cancer cell proliferation and survival and is needed for normal physiological activities, such as heart function and development of the nervous system. How does ErbB2 activation make cancer cells invasive and when? Comprehensive understanding of the cellular mechanisms leading to ErbB2-induced malignant processes is necessary for answering these questions. Here we present current knowledge about the invasion-promoting function of ErbB2 and the mechanisms involved in it. Obtaining detailed information about the “bad” behavior of ErbB2 can facilitate development of novel treatments against ErbB2-positive cancers.

## 1. ErbB2 Function and Basis of the ErbB2-Induced Malignancy

### 1.1. ErbB2 as a Potent Oncogene, Biomarker and a Therapeutic Target

The ErbB2/HER2/Neu receptor belongs to the subclass I receptor tyrosine kinase subfamily, also known as the epidermal growth factor receptor (EGFR) family, which includes three additional members: EGFR/ErbB1, ErbB3, and ErbB4. Overexpression of ErbB2, primarily owing to gene amplification, is observed in many human cancers of epithelial origin including bladder, breast, gastric, lung, ovarian, colorectal, pancreatic, and head and neck cancers [1,2,3,4,5,6,7,8,9,10]. ErbB2 amplification is most common in breast cancer and concerns about 25%–30% of invasive ductal breast carcinomas [11].

In this review we will mainly discuss ErbB2-induced invasion in the context of breast cancer, as ErbB2 function is most extensively studied in breast cancer, where its aberrant overexpression and activation is strongly linked to an invasive, aggressive phenotype and poor prognosis [12,13]. Indeed, ErbB2 amplification is used as an independent prognostic indicator for overall survival and time to relapse in breast cancer, where the increased ErbB2 expression level correlates positively with the presense of cells in S-phase, tumor size, grade, aneuploidy, metastasis to the lymph nodes, and resistance to endocrine therapy [11,13]. In addition to being a reliable biomarker of aggressive and invasive disease, ErbB2 is also a validated therapeutic target in breast cancer. ErbB2 positive breast cancer patients are treated with combination therapy consisting of chemotherapy and trastuzumab (Herceptin), a humanized monoclonal antibody that targets the extracellular NH_2_-terminal domain of ErbB2 [14]. Trastuzumab is most beneficial for patients with ErbB2 positive, early stage breast cancer [15]. It is estimated that about 70%–80% of ErbB2 positive invasive breast cancers are either initially non-responsive or develop resistance to trastuzumab [15,16]. Very few long-term studies exist on the effect of combined trastuzumab and chemotherapy treatment in metastatic breast cancer. A recently published small-scale follow up study of a long term outcome of trastuzumab and chemotherapy treated ErbB2 (HER2) positive metastatic breast cancers demonstrated durable complete response for only 6%–9% of cases [17]. Currently, patients with trastuzumab resistant breast cancers are treated with chemotherapy and lapatinib, a dual pharmacological EGFR and ErbB2 inhibitor that targets the intrinsic kinase domains of these two receptors. Pertuzumab, a humanized monoclonal antibody that also targets the NH_2_-terminal domain of ErbB2 and blocks the ErbB2 dimerization with ErbB3, is recently also accepted for a treatment of ErbB2 positive breast cancer in combination with chemotherapy. Existence of multiple validated drugs such as trastuzumab, pertuzumab, and lapatinib makes it possible to change from one treatment to another upon resistance and relapse increasing the favorable patient prognosis. The benefits of dual targeting of ErbB2 in breast cancer have been recognized and recently dual targeting has been accepted as an additional treatment strategy [18,19].

### 1.2. ErbB Family Connections

The NH_2_-terminal extracellular domain of ErbB2 is structurally different from that of the other family members. Contrasting to those, the extracellular domain of the ErbB2 receptor is constitutively held in a fixed conformation similar to that of the ligand-activated ErbBs rendering it unable to bind ligands [20]. ErbB2 can be activated by dimerization with other, ligand-bound ErbB family members [21,22,23]. Epidermal growth factor (EGF) and EGF–like ligands, amphiregulin, and TGFα are the preferred ligands of EGFR, while ErbB3 and ErbB4 prefer heregulins and neuregulins [21]. Overexpressed, constitutively active ErbB2 homodimers can also form spontaneously in a ligand-independent manner [24] and the high basal kinase activity of ErbB2 allows it to autophosphorylate and signal efficiently even in the absence of a ligand [25]. ErbB2 is the preferred dimerization partner of the other ErbB-family members [23,26] and the ErbB2-containing dimers are the most potent signal amplifiers. This is because ErbB2 binds to a larger set of phosphotyrosine-binding proteins than the other family members [27] and because the endocytotic downregulation of ErbB2 containing dimers is slower and less efficient than those not containing ErbB2 [28,29,30]. Receptor dimerization is the key function that leads to activation of the intrinsic kinase domain, resulting in autophosphorylation of specific tyrosine residues within the cytoplasmic tail of the ErbB2 receptor [31]. The phosphorylated tyrosine residues function as docking sites for signaling molecules, which, dependent on the identity of the ligand and the dimer, activate specific signaling pathways, leading to the correct cellular response [21,22,23,24,25,26,27,28,29,30,31,32,33].

### 1.3. Two Major Functional Forms of ErbB2: p185 and p95

The most common form of ErbB2 is the full-length 185 kD wild type ErbB2 (p185 ErbB2). An alternative form, known as the p95 ErbB2, is often expressed in highly invasive and metastatic breast cancers [16,34,35]. It is a truncated form of ErbB2 that lacks the NH_2_-terminal extracellular domain. P95 ErbB2 is mainly expressed in aggressive breast cancers with lymph node metastases and its expression is an independent prognostic factor for ErbB2-positive breast cancer cases with significantly worse outcome, and predicts resistance to therapeutic ErbB2 inhibition [16,34,36,37,38]. The most common reason for the truncation of ErbB2 is increased extracellular protease activity [39,40,41,42]. This cleavage is most likely performed by ADAM10 metalloprotease resulting in 95–100 kD membrane bound fragment [41,43]. It can also result from alternative splicing or alternative translation initiation which give rise to membrane-bound ErbB2 fragments of 90–95 kD and 100–115 kD [44,45,46]. These truncated products are all biologically active and collectively called as p95 ErbB2 [47]. The p95 ErbB2 can form constitutively active homodimers with ErbB2 independently of ligand binding or heterodimers with ligand bound ErbB3 or ErbB4 [48,49]. The truncated ErbB2 receptor has higher kinase activity than the full-length ErbB2 when measured as autophosphorylation of ErbB2 by immunocomplex kinase assays [50]. Overexpression of p95 ErbB2 in MCF7 human mammary adenocarcinoma cell line results in its enhanced constitutive phosphorylation, appearance of a more malignant morphology with lamellopodia/invadopodia-like elongated cellular protrusions and renders MCF7 cells invasive [48,51,52,53,54]. Overexpression of p95 ErbB2 in mouse mammary gland under the mouse mammary tumor virus (MMTV) promoter facilitates mammary tumor metastasis to lungs *in vivo* over the full-length ErbB2 [48,55]. The facilitated lung metastasis formation of p95 ErbB2 expressing cells compared to full-length ErbB2 expressing cells is contributed by the p95 ErbB2-induced senescence secretome that contains several pro-tumorigenic factors that are capable of promoting metastasis in mice [56].

Overexpression of p95 ErbB2 increases the expression of endogenous EGFR and prolongs its EGF-induced activation [16,48,51]. Overexpression of p95 ErbB2 leads to the activation of phospholipase C gamma (PLCγ) and mitogen activated protein kinase (MAPK/ERK), Src, protein kinase B (PKB/Akt), Janus kinase/signal transducer activator of transcription (JAK/STAT), and stress-activated protein kinase/c-Jun N-terminal kinase (SAPK/JNK) pathways just as the overexpression of the full length ErbB2 but with higher magnitude [48,51,52,55]. The endogenous p95 ErbB2 in BT474 breast cancer cells does not respond to EGF as the full-length ErbB2, but instead gets phosphorylated and activated in response to ErbB3 and ErbB4 ligand heregulin [49]. Supportively, p95 ErbB2 forms preferentially heterodimers with ErbB3 over EGFR in BT474 tumor xenografts [49]. Depletion of EGFR, on the contrary to the depletion of ErbB2, ErbB3 or ErbB4 from p95 ErbB2 overexpressing MCF7 cells, has no effect of the p95 ErbB2-induced cysteine cathepsin activity, a key proteolytic activity that is needed for their invasion in Matrigel in 3-dimensional invasion assays [52]. This altogether suggests that p95 ErbB2, even though can increase the endogenous EGFR activity, most likely preferentially dimerizes and transmits its invasion supporting signals via ErbB2, 3, or 4, in MCF7 and BT474 cells. Moreover, ErbB3 is more abundantly expressed than EGFR in normal breast tissue as well as in a vast majority of breast cancer cell lines including MCF7 and BT474 cells [57], making it a more likely dimerization partner for ErbB3 than EGFR.

The main clinical problem associated with the p95 ErbB2 is that it is more potent oncogene than the full-length ErbB2, but it does not respond to trastuzumab-based therapy, as it is missing the trastuzumab-binding site [16,58]. It is estimated that approximately 20%–30% of ErbB2-positive primary breast tumors express the truncated ErbB2 [35,59]. Although lapatinib can efficiently target p95 ErbB2 [59], unfortunately, expression of p95 ErbB2 is also involved in the acquired therapeutic resistance to pharmacological ErbB2 tyrosine kinase inhibitors including lapatinib with a mechanism that is not completely understood [36].

### 1.4. ErbB2 Downstream Signaling and its Physiological and Cellular Responses

ErbB2 is an important physiological signal transducer that has shown to play essential role in the regulation of cell proliferation, differentiation, survival and migration during embryonic development and in tissue maintenance in adults. ErbB2 is expressed in multiple organs and its activation is essential for various physiological processes such as oligodendrocyte differentiation and myelin formation during brain development, establishment of radial glia in the cerebral cortex, cardiogenesis, development of mammary gland, maintenance of muscle spindle, and prevention of cardiomyophaty in the adult heart [60,61,62,63,64,65,66,67]. In a normal, healthy organism, ErbB2 activation and function are tightly regulated. The essential role of ErbB2 in normal cardiac function is demonstrated by ErbB2 knockout mice and by therapeutic targeting of ErbB2 in cancer. Genetic inactivation of ErbB2 is lethal at embryonic day 10.5 due to impaired cardiac and neural development [60]. Cardiac myocyte-specific conditional ErbB2 knockout mice develop cardiomyophaty and the cardiomyocytes isolated from these mice are sensitive to anthracycline administration [68], which may be partially due to mitochondrial dysfunction caused by ErbB2 inhibition [69]. Supportively, heterozygous Neuregulin 1 knockout mice expressing low levels of this physiological ErbB3 and ErbB4 ligand are susceptible for doxorubicin-induced heart failure [70]. Similarly, in human, ErbB2 inhibition by trastuzumab can have cardiotoxic side effect [71]. Especially, this has been attributed to combination of trastuzumab and anthracycline-containing chemotherapy, where trastuzumab enhances the incidence of anthracycline-mediated cardiac systolic dysfunction [72]. As with trastuzumab, similar level of cardiotoxicity is also detected for pertuzumab but not for lapatinib that shows less adverse cardiac effects in clinical use than the two antibodies [18,73]. This could be because lapatinib inhibits ErbB2 kinase activity without affecting ErbB3 or 4 and trastuzumab and pertuzumab inhibit ErbB2 dimerization, thus also indirectly affecting ErbB3 and ErbB4 function.

The cellular outcome of the ErbB2-mediated signaling is controlled by availability of the ligand, the cellular receptor composition, availability of signaling mediators and the downregulating processes such as the rate of receptor internalization and transport to the endosomal compartment. The ErbB2 activated downstream signaling pathways include several well-studied signaling pathways as discussed above and reviewed elsewhere [32,74,75]. At the cellular level, ErbB2 overexpression increases proliferation by causing deregulation of G1-S transition by modulating the activation status of Cyclin E-Cdk2 complex and promoting the expression of Cyclin D via c-Myc. It also controls the expression, localization and activity of the cyclin-dependent kinase inhbitors p27Kip1 and p21cip1/Waf1 increasing cell proliferation [76,77,78,79,80]. In addition to proliferation, ErbB2 signaling promotes cell survival via Akt/PKB pathway by inducing NFKB levels both in breast cancer cells and in mammary tumors derived from MMTV-Neu mice [81].

However, perhaps one of the most important ErbB2-induced cellular change contributing to invasiveness is the increased motility. ErbB2 signaling via Ras and PI3K activates the Rho GTPases Rac and cdc42, which are essential regulators in cytoskeleton rearrangement and cell motility [82]. Motility is also increased via ERK-mediated activation of myosin light chain kinase (MLCK) leading to increased myosin light chain (MLC) phosphorylation and motor activity [83]. ErbB2-mediated activation of PLCγ resulting in PIP2 hydrolysis leads to release of gelsolin from the plasma membrane, promoting formation of lamellopodia and increasing cell motility [82]. The increased motility of p95 ErbB2 expressing MCF7 cells is at least partially mediated via cortactin phosphorylation [53]. Cortactin is a cytoskeleton-binding protein, in which phosphorylation at Tyr 421, in response to p95 ErbB2 signaling, localizes it into invadopodia-like cellular protrusions and increases cell motility [53]. Cortactin is phosphorylated by ERK and by Src, the latter being the major cortactin kinase [53]. Overexpression of ErbB2 or the p95 ErbB2 in MCF7 cells induces cortactin expression [48] and, thus, cortactin may be involved in the migration induced by the full-length ErbB2 as well. Cortactin, which is used as a marker of invadopodia, is also required for CD44 and EGF induced cell migration [84,85,86].

## 2. Invasive Signaling of ErbB2

### 2.1. ErbB2 is not Enough

**Figure 1:**
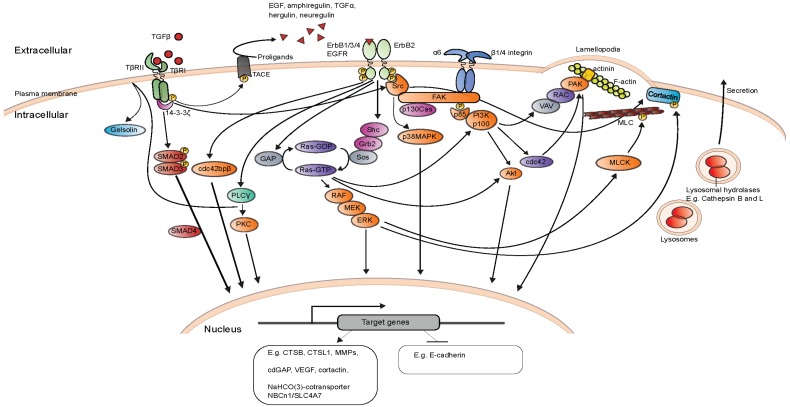
Simplified presentation of invasion and migration inducing molecular mechanisms and signaling pathways, mainly based on studies done with ErbB2 and p95 ErbB2 expressing MCF7 and MCF10A cells [21,48,52,53,82,83,89,90,97,98,99,100,101,102,103,104,105,106].

The most explored ErbB2-activated signaling pathways are best known for their capability to regulate cell growth, survival, migration and cell cycle progression. The efficient inhibition of ErbB2-induced proliferation and tumor growth was also a central qualification in the development of trastuzumab [15]. Invasion and metastasis are however the most fatal activities connected to ErbB2. Several studies have been carried out to understand the molecular mechanism involved. The molecular mechanisms identified utilizing cultured MCF7 and MCF10A cells are summarized in Figure 1. Aberrant ErbB2 activation is found in about 25%–30% of invasive ductal breast carcinomas, however, up to 85% of mammary ductal carcinomas *in situ* (DCIS), which are early non-invasive mammary carcinomas, also overexpress ErbB2 [87,88]. This indicates that the mere increase in ErbB2 expression is not sufficient to induce metastatic breast cancer. Supportively, overexpression of ErbB2 in immortalized breast epithelial MCF10A cell line grown as 3-dimensional Matrigel cultures induces formation of “growth arrested” 3-dimensional structures, e.g., acini that resemble normal mammary acini located in the terminal ductal lobules of human breast with no sign of invasion [89,90]. The high incidence of ErbB2 positive non-invasive DCIS together with multiple studies showing the incapability of ErbB2 to induce invasion of non-cancerous MCF10A mammary epithelial cells have led into a “two-hit” theory of invasion, where overexpression of the ErbB2 oncogene is the first hit, which will only promote invasive cancer after receiving the second, additional hit. Supporting the “two-hit” theory, EGF treatment of ErbB2 overexpressing, non-invasive MCF10A cells activates. TGFβ signaling leads to their invasion in 3-dimensional Matrigel invasion assays [90]. It is important to note that although the “two-hit” theory for ErbB2-induced invasion is well experimentally supported, it is still not known if ErbB2 positive invasive ductal carcinomas develop from ErbB2 positive DCIS.

In a large number of independently established murine models of ErbB2-positive breast cancer, mammary gland specific expression of ErbB2 or Neu (rat ErbB2) alone is sufficient to produce metastatic mammary tumors, however with variable latency, which is most likely due to differences in the integration site and the copy number [91]. In mice, overexpression of ErbB2/Neu seems to be enough for the induction of metastatic mammary cancer. Here the second hit could result from growth factor release as has been shown for the MCF10A cells expressing ErbB2 [90]. Another, perhaps more likely reason is that in the vast majority of the invasive murine tumors, transgenic ErbB2/Neu gains activating mutations in its extracellular domain [92,93,94] indicating a strong selective mutational pressure in mice [91]. Interestingly, somatic activating mutations of ErbB2 have very recently been found in some human cancers lacking ErbB2 amplification [95]. These are, however, totally missing from breast cancers harboring ErbB2 amplification [96].

### 2.2. Transforming Growth Factor β and ErbB2-Induced Invasion and Metastasis

Transforming growth factor (TGF) β is a secreted polypeptide that functions as a potent tumor suppressor eliciting its downstream signaling by binding to a heteromer of TGFβ type I and type II receptors (TGFβRI and TGFβRII) that are transmembrane serine/threonine kinases. In normal healthy epithelia TGFβ signaling induces apoptosis and inhibits cell proliferation. In transformed epithelia TGFβ shifts from its tumor suppressor role to oncogenic and tumor supportive [107,108]. The first indication of the cooperation between ErbB2 and TGFβ in mammary cancer comes from an *in vivo* study where MMTV-Neu (expressing rat ErbB2 under MMTV promoter) mice are crossed with MMTV-TGFβ mice expressing constitutively active, mutated TGFβ [109] or TGFβRI [110]. The mice expressing both Neu and constitutively active TGFβ tumors that are more invasive and of higher histological grade and they also have more circulating tumor cells, and more lung metastasis, than MMTV-Neu only mice [109]. Detailed characterization of the tumors reveled that the bigenic mice have higher levels of activated phosphor forms of Smad2, Akt1, ERK-MAPK, and p38-MAPK, as well has higher levels of vimentin and active Rac1 than Neu only mice [109]. Supportively, expression of a dominant negative TGFβRII decreased and expression of two different dominant active TGFβRI mutants increased the incidence of lung metastasis in mice expressing various forms of Neu under MMTV promoter [110,111]. The data altogether confirming that TGFβ signaling amplifies oncogenic ErbB2 signaling *in vivo* and further promotes invasion and metastasis of ErbB2 positive cancer cells.

An independent study was carried out to understand at a cellular level why and how most of the ErbB2 positive DCIS do not develop into invasive and metastatic disease. To assess the role and function of ErbB2 overexpression in the development of invasive breast cancer, ectopic expression of a recombinant p75.B2 ErbB2 construct was studied in MCF10A cells [89]. This construct consists of the extracellular transmembrane domain of the p75 low-affinity nerve growth factor (NGF) receptor (p75NGRF) and the cytoplasmic domain of ErbB2 linked to the synthetic-ligand-binding domain of FK506-binding protein (FKBP). Importantly, this recombinant ErbB2 can be induced to dimerize independently of endogenous ErbB ligands by addition of the FKBP ligand AP1510. The expression and induction of recombinant ErbB2 induced proliferation of mammary acini in 3-dimensional Matrigel cultures without rendering cells invasive [89]. Screening of 30 pre-selected full-length cDNAs revealed TGFβ (TGFβ) as a factor that could induce migration and invasion of recombinant ErbB2 expressing MCF10A cells [89]. Co-operation of the ErbB2 and the TGFβ activated MAPK/ERK signaling pathway induced and supportively, inhibition of ERK activity inhibited, their co-operative effect on cell migration, suggesting for a central role for MAPK/ERK in this process [89]. A corresponding independent study verified the role of the MAPK/ERK pathway in migration and invasion and identified p38 and Akt as additional kinases further activated by the ErbB2-TGFβ co-operation [97].

### 2.3. More Players for the Game

Soon more studies were carried out to understand the co-operative function of ErbB2 and TGFβ. One central mechanism by which TGFβ signaling amplifies ErbB2 signaling is by increasing the availability of the ErbB family ligands. TGFβ signaling in ErbB2 expressing MCF10A cells increases EGFR ligand shedding by a mechanism that involves phosphorylation of TNFα converting sheddase enzyme (TACE/ADAM17) [98] and is independent of Smad [99]. TACE/ADAM17 is a metalloprotease that is responsible for shedding of many cell surface proteins. Its TGFβ-induced translocation to the cell surface in ErbB2 overexpressing cells induces secretion of TNFα, amphiregulin and heregulin facilitating ErbB2 downstream signaling [99].

One important ErbB2-activated, invasion-promoting group of signaling proteins is the p21-activated protein kinase (PAK) family of serine/threonine kinases that function as effectors of Cdc42 and Rac and are involved in cytoskeletal organization and cancer cell invasion in general [112,113]. In MCF10A cells the co-expression of TGFβ and ErbB2 induces ErbB2 translocation to lamellopodia via mechanism that is PI3K dependent and involves Rac1 and PAK1 kinase activation and reorganization of the actin cytoskeleton [97,100]. Moreover, TGFβ induces recruitment of a large protein complex containing Rac1 and PAK1, ErbB2, Vav2, actin, and actinin complex to lamellopodia. This recruitment can be abrogated by inhibition of any of the kinases involved either pharmacologically or by overexpressing dominant negative forms as well as by RNA interference based depletion of actinin [100]. Mere overexpression of activated PAK1 or Rac1 in MCF10A cells increases proliferation and distorts the normal acinar structure [114]. In addition to PAK1, the other PAK family members, PAK4, 5, and 6 are implicated in ErbB2-induced malignancy of breast cancer cells [52,115]. In TGFβ and ErbB2 co-expressing MCF10A cells, clustering of ErbB2 with integrins α6, β1, and β4 is focal adhesion kinase (FAK/PTK2)-dependent and important for their migration [99]. This process is mediated by ligand-induced EGFR activation leading to subsequent phosphorylation and activation of protein tyrosine kinases Src and FAK. Inhibition of Src-FAK complex formation by FAK siRNA abolishes the TGFβ-induced invasion of ErbB2 expressing MCF10A cells in transmembrane invasion assays [99]. Supporting the importance of integrins in this process, treatment with an inhibitory antibody against β1 integrin or integrin α6 siRNA treatment abrogates the TGFβ-induced motility of the ErbB2 expressing MCF10A cells [97].

### 2.4. Alternative Approaches to Identify Invasion Mechanisms

Recently, a non-biased whole kinome siRNA screen was carried out to identify kinases that regulate p95 ErbB2-induced malignancy utilizing MCF7 cells expressing 95 kD ErbB2 mimicking plasmid p95ΔN-ErbB2 [51,52]. Induction of p95ΔN-ErbB2 expression made cells aggressive and capable for invading, overnight, in 3-dimensional Matrigel invasion assays [52]. It also made lysosomes translocate from their normal perinuclear positions to ErbB2 expression-induced lamellopodia-like cellular protrusions. Invasion was strictly dependent on a modest ErbB2-induced increase in the cysteine cathepsin B and L expression and activity [52]. Cathepsins B and L are lysosomal cysteine cathepsins that upon secretion to the extracellular space can cleave and activate urokinase plasminogen activator, heparanase, and various matrix metalloproteases as well as E-cadherin and, thus, contribute to invasion and metastasis [116,117]. Utilizing the ErbB2-induced cysteine cathepsin activity as readout, a whole kinome siRNA library screen was performed to identify kinases whose depletion decreased the cysteine cathepsin activity [52]. In the study, cdc42 binding protein kinaseβ, PAK4, 5, and 6, and PKCα were identified as novel essential mediators of ErbB2–induced cysteine cathepsin expression, activity and invasion in 3-dimensional Matrigel cultures. Additional positive hits included ERK2, TGFβRI, and TGFβRII, as well as ErbB2, 3, and 4. All the same kinases were also found central for the invasiveness of the ErbB2 positive MDA-MB-453 and SK-BR-3 breast cancer cells endogenously expressing high levels of the full length ErbB2 [52]. Transcription factors MZF1 and Ets1 were identified as targets of this invasive signaling directly regulating the increased expression of *CTSB* and *CTSL1* (cathepsin B and L) respectively [52]. Multiple studies report that increased cathepsin B and L expression and activity correlate positively with and are involved in the invasion and metastasis of several cancers [118]. Supportively, both *CTSB* and *CTSL1* mRNA and their protein levels also correlate positively with ErbB2 status in primary breast cancer [52]. A very important result of this work is the notion that an unbiased kinome screen could still identify several novel kinases as regulators of ErbB2-mediated invasion thus giving new possibilities to control and explore the ErbB2-induced invasiveness downstream of the receptor. Another paramount observation was that modest changes in the expression of key molecules, such and cathepsin B and L could have major effect for invasion.

High levels of 14-3-3ζ and ErbB2 with positive lymph node status increase the risk for the development of metastatic breast cancer [119]. Regulatory protein 14-3-3ζ is a member of the 14-3-3 family that is involved in binding and regulation of the activity of many signaling proteins. A study utilizing MCF10A cells overexpressing ErbB2 and 14-3-3ζ together or separately showed that both of them are needed for the invasion of MCF10A cells into 3-dimensional Matrigel cultures [101], suggesting that overexpression of 14-3-3ζ can overcome the need of TGFβ in ErbB2-induced invasion. In that study Src was shown to mediate the invasive signaling in the ErbB2 and 14-3-3ζ overexpressing MCF10A cells. Moreover, these cells exhibited clear loss of epithelial cell marker, E-cadherin, indicating that they had most likely undergone epithelial-mesenchymal transition (EMT). Interestingly, on the contrary for the corresponding ErbB2 and TGFβ overexpressing cells, Rac1 and PI3K were not involved in the invasion of the ErbB2 and 14-3-3ζ overexpressing cells. The loss of E-cadherin expression was shown to occur with a mechanism whereby 14-3-3ζ activates the TGFβ /Smads pathway leading to upregulation of the zinc-finger transcription factor ZFHX1B that binds to E-cadherin promoter and represses its expression [101] indicating that 14-3-3ζ is activating the same invasion-promoting downstream responses than TGFβ.

### 2.5. Invasion and Metastasis Inducing Targets of ErbB2 Downstream Signaling

Several expression array analyses have been carried out in order to identify the genetic signature activated by ErbB2 that defines the malignancy of ErbB2 in breast cancer. Multiple and extensive large-scale expression array, mRNA sequencing and proteome studies exist of primary breast tumors that have led for example into defined molecular subtyping of breast cancer. These studies will not be discussed in this review, since, as on their own, they do not give clear information of the mechanistic processes needed for ErbB2-induced invasion and metastasis. Perhaps the most helpful of the expression array and proteome analyses in terms of understanding the mechanism of the ErbB2-induced invasion could be the studies utilizing ectopic, inducible overexpression of ErbB2 or p95 ErbB2 in MCF7 cells [48,120,121], where overexpression of ErbB2 is enough to make cells invasive or those using stably ErbB2 overexpressing normal breast epithelial cells, such as MCF10A that are induced to invade via activation of EGF/TGFβ signaling or by expressing the aggressive truncated p95 ErbB2 [55,90,122]. In principle, both model systems could allow the detection of the earliest transcriptional changes in response to ErbB2 overexpression and activation.

Expression array analysis of MCF7 cells expressing either inducible ErbB2 or p95 ErbB2 revealed that the expression of p95 ErbB2, resulted in similar transcriptional response, but in a shorter time frame, indicating different kinetics and supporting the observations of p95 being more oncogenic than the full length ErbB2 [48]. This phenomenon has also been verified in *in vivo* studies comparing these two forms of ErbB2 [48,55]. Increased expression of the adhesion molecules PHLDA1, EPHA2, and integrin β1 were identified in both cases, whereas integrin α5 was only upregulated by ErbB2 and Met, MMP-1 and integrin α2 were only upregulated by p95 ErbB2 [48]. With this work, a 76-gene signature was established consisting of genes that were upregulated more than three-fold in p95 expressing cells and less than two-fold in full-length ErbB2 expressing cells. In addition to Met, MMP-1 and integrin α2 this signature contained several genes generally involved in cancer progression and metastasis, such as IL-11, TGFα, and EGFR [48]. Analysis of a limited group of primary breast tumors identified a small, but significant, 76-gene signature group of patients with decreased survival rate [48]. A very recent independent study comparing the proteomes of MCF7 cells stably overexpressing ErbB2 to corresponding control cells reported 231 differentially expressed proteins of which 85 were upregulated and the rest downregulated by ErbB2 [121]. Interestingly, when compared to [48] this study identified only four common targets in ErbB2 expressing MCF7 cells: anterior gradient homolog 2 (AGR2), dehydrogenase reductase (SDR) family member 2 (DHRS2), glutathione S-transferase M3 (GSTM3), and isochorismatase domain containing protein 1 (ISOC1).

Recently, another study was carried out to identify invasive target genes by using ErbB2 overexpressing MCF10A cells that were induced to invade by EGF treatment [90]. Even though EGFR is not a strong oncogene in breast cancer and the overexpression of EGFR cannot induce mouse mammary cancer by itself [123], it can co-operate with ErbB2 in MCF10A cells making them invasive in 3-dimensional Matrigel assays [124]. Comparative transcriptomics analysis between the EGF-induced, ErbB2 expressing MCF10A cells and a set of ErbB2 expressing invasive ductal carcinoma (IDC) samples identified a shared signature of 361 genes [90]. This EGF-activated invasive signature contained several components of the TGFβ signaling pathway making it the most significantly enriched pathway in both cases. Supportively, knockdown of TGFβ downstream signaling component Smad4 in these cells blocked their invasiveness, indicating that EGF-treatment activated TGFβ signaling [90]. One of the ErbB2 upregulated gene and protein was extracellular matrix modifying enzyme lysyl oxidase-like 2 (LOXL2) [90], of which mere overexpression can render MCF10A cells invasive by inducing ErbB2 activity with a mechanism that involves LOXL2-induced production of reactive oxygen species [125]. Interestingly, overexpression of p95 ErbB2 in MCF10A cells could bypass the TGFβ and ErbB2 co-expression requirement for MCF10A invasion in 3-dimensional Matrigel cultures [55]. In this study the culture media was supplemented with EGF, which may have contributed to the invasion. It is likely that p95 ErbB2, which is a more potent oncogene than ErbB2 itself, can induce invasion with smaller amounts (or none) of supportive EGF/TGFβ signaling.

As all of these studies identified changes in the expression of multiple genes and proteins, a common way of handling the results has been to focus on well-known mediators of invasion and migration that undergo the biggest changes, such as cell adhesion molecules and proteases. The most surprising result of all of these studies is that in each case the set of the most up- and downregulated genes/proteins is different and very little overlap in the target lists of different research groups can be detected even though the cellular phenotype is the same. This could for example be due to differences in tissue culture conditions, cells, timing, ErbB2 integration site, or expression levels in different laboratories. All these variable nominators could induce “dramatic” temporary changes in the gene expression patterns most of which would not necessary affect their invasiveness. It is also noteworthy that not all ErbB2 downstream signaling activates invasion and metastasis programs. Thus, it is likely that only a small fraction of genes with minor expression changes may be the most critical ones. Moreover, it is also possible that many of the important changes contributing to invasion can simply just be posttranslational modifications occurring in existing proteins.

### 2.6. Other Important Mediators and Targets of ErbB2-Induced Invasion

Cancer cell invasion requires activation of proteases that can degrade extracellular matrix. ErbB2 overexpression induces expression of cysteine cathepsins B and L leading to their increased activity, which is necessary for ErbB2-induced invasion of ErbB2 and p95 ErbB2 expressing breast cancer cells [52]. Cathepsin B and L are secreted from cancer cells to the extracellular space where they are directly involved in the proteolytic cleavage and activation of heparanase, matrix metalloproteases (MMP), and urokinase plasminogen activator (uPa), which are important mediators of extracellular matrix degradation and actively involved in cancer invasion and metastasis [116,117]. Ectopic expression of ErbB2 in MCF10A cells increases the expression of MMP-2 [102] and overexpression ErbB2 in MCF7 cells induces expression and secretion of MMP-9 and uPA [103], in a transcriptional process that can be potentiated by ectopic expression of Ets1 or Ets2 [126]. The basal levels of MMP-1 and MMP-9 are increased in various breast cancer cells, including MCF7, in response to heregulin-β1 via activation of MAPK/ERK pathway [127] and overexpression of Ets1 in ErbB2 expressing breast cancer cells increases the expression of MMP-1 [128]. One potentially important p95 ErbB2 upregulated protein in MCF7 cells is the Na(+),HCO(3)(-)-cotransporter NBCn1/SLC4A7 that is involved in the acid extrusion and acidification of the extracellular space [104,105]. Moreover, overespression of p95 ErbB2 activates the Na^+^/H^+^ exchanger NHE1 resulting in its localization to invadopodies contributing to the acidification of the extracellular space [54]. Acidic tumor microenvironment supports extracellular activity of cysteine cathepsins B and L, which, in turn, activate uPa and MMPs [129].

### 2.7. ErbB2 Overexpressing Mice and ErbB2-Induced Metastasis

*In vivo* studies with murine mammary gland directed overexpression of MMTV-ErbB2/Neu and MMTV-TGFβ or constitutive active mutant TGFβR show that activation of TGFβ signaling pathway enhances ErbB2-induced tumor growth and metastasis to lungs, which can otherwise be a rather slow process [91]. In addition to ErbB2, several studies using mammary gland directed overexpression of polyoma middle-T antigen (PymT) to model metastatic breast cancer has been carried out. Mere overexpression of PymT in mammary gland induces partially same signaling pathways than ErbB2/Neu, resulting in tumors that metastasize rapidly to lungs [130]. Here, we will only present studies involving overexpression of ErbB2/Neu.

Studies combining ErbB2/Neu with overexpression or knockout of genes of interest have identified several different genes that can contribute to ErbB2-induced metastasis. One of these is vascular endothelial growth factor (VEGF) that is a signaling molecule inducing angio- and vasculogenesis, two processes that are very important for tumor growth and metastasis. As expected, bigenic expression of activated MMTV-Neu and MMTV-VEGF results in increased mammary gland vascularization, mammary tumor growth acceleration and increased lung metastasis [131]. Transgenic overexpression of mutated constitutively active Stat3 under the MMTV promoter together with MMTV-Neu induces Neu-mediated metastasis by upregulation of focal adhesion protein Cten [132] and genetic inactivation of a Stat3-responsive oncogene Bcl3 from MMTV-Neu mice dramatically reduces Neu-induced lung metastasis [133]. Interestingly, bigenic expression of activated MMTV-Neu and constitutively active Akt1 under MMTV promoter results in accelerated tumor onset but suppressed metastasis [134], implicating that the malignancy-promoting function of Akt1 in mammary cancer is mainly targeting the primary tumor. Knocking out phosphatase and tensing homolog (PTEN), which is a tumor suppressor that is inactivated in 40% or ErbB2-positive breast cancers [135], increases angiogenesis and metastasis from ErbB2 overexpressing mammary gland [136]. Similarly, loss of signaling protein 14-3-3σ from MMTV-ErbB2 mice [137] and loss of cell adhesion protein α1β1 integrin from MMTV-Neu mice [138] enhances ErbB2/Neu induced metastasis. On the contrary to α 1β1 integrin, loss of β4 or β1 integrin function results in decreased lung metastasis [139,140] indicating differing functions for integrin family members in this process. The invasion facilitating function of β4 integrin is mediated by Stat3 [140], of which activation in MMTV-Neu overexpressing mice also increases lung metastasis [132]. Lack of expression of lipocalin 2, which is a secreted glycoprotein and involved in MMP-9 stability, delays ErbB2-induced mammary tumor metastasis with a mechanism that involves decreased MMP-9 activity [141]. Loss of expression or function of several signaling molecules inhibits lung metastasis of mammary tumors in ErbB2/Neu overexpressing mice. These include protein tyrosine phosphatase 1B (PTP1B), adapter protein Gab2, EphA2 receptor tyrosine kinase, Rho GTPase activating protein p190B, receptor activator of nuclear factor- KB (RANK), estrogen receptor α, semaphorin receptor plexin-B1, and Rac specific guanidine nucleotide exchange factor DOCK1 [142,143,144,145,146,147,148,149], underlining the role of several signaling pathways in ErbB2-induced invasion and metastasis.

In addition of verifying a role of a specific molecule in invasion *in vivo*, murine models give important information on cancer metastasis and tumor-induced angio- and vasculogenesis and help dissecting the stromal influence to these processes. Metastasis is a very complex organ-specific process that involves variety of orchestrated communications and interactions between the tumor and the host, all of which can only be studied in *in vivo* models. All these studies have shed light to the role of specific molecules in the ErbB2-induced invasion and metastasis and resulted in many important observations. However, the vast variety of the used ErbB2/Neu mutants and expression constructs have resulted in a variable latency and differing efficiency with which the tumors and metastases appear making it challenging to make detailed comparison of the results [91,150].

### 2.8. ErbB2, Cancer Initiating Cells and EMT in Breast Cancer Invasion

Epithelial-mesenchymal transition (EMT) is a highly coordinated developmental process where cells of epithelial origin transform into mesenchymal-like cells [151,152]. Oncogenic EMT is a less orchestrated event whereby epithelial cells lose their polarity and adhesion ability, undergo cytoskeletal remodeling, and become motile and invasive. Oncogenic EMT can contribute to cancer progression by allowing malignant cells to invade and form metastases. The role and function of EMT in invasion and metastasis *in vivo* is still a matter of debate especially in high- and low-grade epithelial cancers, such as breast cancer, where distant metastases are histologically similar to the primary tumor, without clear signs of collective EMT [153,154,155]. This implicates that the breast cancer invasion and metastasis may actually occur without EMT, or that standard pathological examination cannot reliably identify single disseminating cells. Thus, a small number of tumor cells may transiently undergo EMT for invasion or, as likely, breast cancer cells may invade independently of EMT [154]. The lack of signs of EMT in sites of metastasis can be explained by a process known as the mesenchymal to epithelial transition (MET) [156], whereby the disseminating tumor cells adapt epithelial morphology upon their metastatic spread into distant organs.

Several studies have linked ErbB2/Neu overexpression to loss of E-cadherin, a process that is very central for EMT in cancer cells [150]. The strongest connector of ErbB2 to EMT is TGFβ. TGFβ is also a powerful regulator of EMT in various cancer cells of different origin, including breast [106]. TGFβ signaling enhances ErbB2 signaling and is often activated in ErbB2-positive malignant cancer. Focal adhesion kinase (FAK) has an important role in the invasion promoting cytoskeleton remodeling induced by ErbB2 and TGFβ in breast cancer cells [98,99,157]. Another important signaling pathways contributing to EMT are the MAPK/ERK and the Wnt/β-catenin pathways that co-operate with TGFβ to induce EMT [158]. Although the direct connection between ErbB2 and Wnt may be missing, elevated levels of the oncogenic Met receptor tyrosine kinase, which is often upregulated in ErbB2 positive breast cancers with poor prognosis, induces Wnt and EMT signatures [159]. Interestingly, ErbB2 activation by constitutive active Raf-1 in breast cancer cells is implicated in MET, the antagonistic process of EMT [160], further complicating the role of the ErbB2 in this process.

Both ErbB2 and TGFβ activation is associated to breast cancer stem cells/ tumor initiating cells. The molecular events controlling EMT and stemness of breast cancer cells are overlapping [161,162,163]. Tumor initiating, circulating tumor cells (CTC) have a central role in the metastatic spread. Isolation of CTCs from a small group of patients with ErbB2-positive metastatic breast cancer revealed that their CTCs had EMT features [164]. The expression of ErbB2 and the stem cell marker ALDH correlate positively in human primary breast cancer [165]. The ectopic expression of ErbB2 in non-invasive MCF7, SUM149, or SUM159 breast cancer cell lines increases the proportion of CSC’s and invasion *in vitro* and facilitates mammosphere formation and appearance of ductal structures in primary tumors in immunocompromised NOD-SCID mice [166], however inducing only occasional metastasis *in vivo* [167]. Interestingly, shRNA-based depletion of PTEN that is inactivated in 40% of ErbB2 positive breast cancers [135], results in further enrichment of CSC population and generation of larger mammary tumors with extensive metastasis to lymph nodes, liver, and lung [167]. In principle, expression of ErbB2 in breast CSCs suggests that ErbB2-targeted anti-cancer therapies would be efficient also against the tumor initiating CSC. However, loss of PTEN is involved in the resistance against ErbB2-targeted therapies and depletion of PTEN from ErbB2 positive CSC generates trastuzumab resistant metastatic tumors in immunocompromised mice [167], anticipating similar problems in the therapy response in human as well.

## 3. Conclusions

ErbB2 is a potent oncogene whose overexpression is notoriously associated with invasive and metastatic cancer. It is especially prevalent in breast cancer where the therapeutic targeting of ErbB2 is well developed consisting of multiple treatment options. Therapeutic ErbB2 inhibition is most beneficial at the early stages of breast cancer but much less efficient against invasive cancer that has already metastasized. Why it is so, is not fully understood, even though some nominators contributing to resistance to ErbB2-targeted treatments have already been identified including appearance of the NH2-terminally truncated p95 ErbB2 and the loss of PTEN. One attractive way to overcome these obstacles could be to inhibit the ErbB2 downstream signaling events that specifically activate invasion and metastasis. Of special interest would be the targetable regulatory molecules such as enzymes and/or their possible regulatory microRNAs. Identification of the signaling components and other key molecules involved in ErbB2-induced invasion and metastasis is ongoing and has already resulted in interesting and potentially meaningful findings as discussed in this review. Comprehensive and detailed understanding of the invasion mechanisms regulating metastatic spread of cancer cells is well on its way and can be expected to open new possibilities to control this fatal activity.
